# Ciliary Body Schwannoma: A Case Report and Literature Review

**DOI:** 10.18502/jovr.v17i4.12339

**Published:** 2022-11-29

**Authors:** Haythem El Mokh, Néjib Ben Yahia, Mustapha Bouziri, Walid Gattoufi, Abdelmajid Khabir

**Affiliations:** ^1^Department of Pathology, Habib Bourguiba University Hospital, Medenine, Tunisia; ^2^Private Laboratory of Pathology, Houmt Souk Djerba, Tunisia; ^3^Ophthalmologist, Houmt Souk Djerba, Tunisia; ^5^https://orcid.org/0000-0003-0031-2242; ^6^https://orcid.org/0000-0001-8122-5110

**Keywords:** Antoni A and B Patterns, Ciliary Body, Local Excision, Schwannoma

## Abstract

**Purpose:**

To present a case of intraocular schwannoma arising from the ciliary body with description of histological and immunophenotypic characteristics.

**Case Report:**

A 32-year-old woman who was followed for glaucoma of the left eye and chronic renal failure at the stage of hemodialysis presented with buphthalmos and two weeks of blurry vision of the left eye. A magnetic resonance imaging exam was performed suspecting melanoma. Enucleation was rapidly performed. The histological examination after HE (Hematoxylin and Eiosin) and HEA50 (Hematoxylin and polychromatic solution EA 50) staining showed proliferation of mesenchymal monomorphic fusiform cells with eosinophilic cytoplasm and small oval nuclei which showed a tendency toward palisading. Some parts of the tumor were hypercellular with a fascicular arrangement (Antoni A pattern); other parts were weakly cellular with a myxoid arrangement (Antoni B pattern). Several Verocay bodies and a lot of hemorrhagic suffusions were described. Mitotic ﬁgures were very rare. Immunohistochemistry staining showed that tumor cells were positive for PS100 and vimentin.

**Conclusion:**

Although ciliary body schwannoma is extremely rare, it should be considered in the differential diagnosis of intraocular tumors.

##  INTRODUCTION

Schwannoma is an uncommon benign peripheral nerve neoplasm that grows slowly and can occur in any location.^[1]^ Intraocular schwannomas represent approximately 1% of the globe tumors.^[2]^ In most cases, the diagnosis is histological after enucleation. Here, we report histological, immunohistochemical findings of a benign ciliary body tumor treated by enucleation as malignancy was suspected, but microscopic examination of the specimen revealed a benign ciliary body schwannoma.

##  CASE REPORT 

A 32-year-old North-African woman presented with two weeks of blurry vision of the left eye. This patient was suffering from chronic renal failure at the stage of hemodialysis and was recently followed for one year for glaucoma of the left eye.

**Figure 1:**
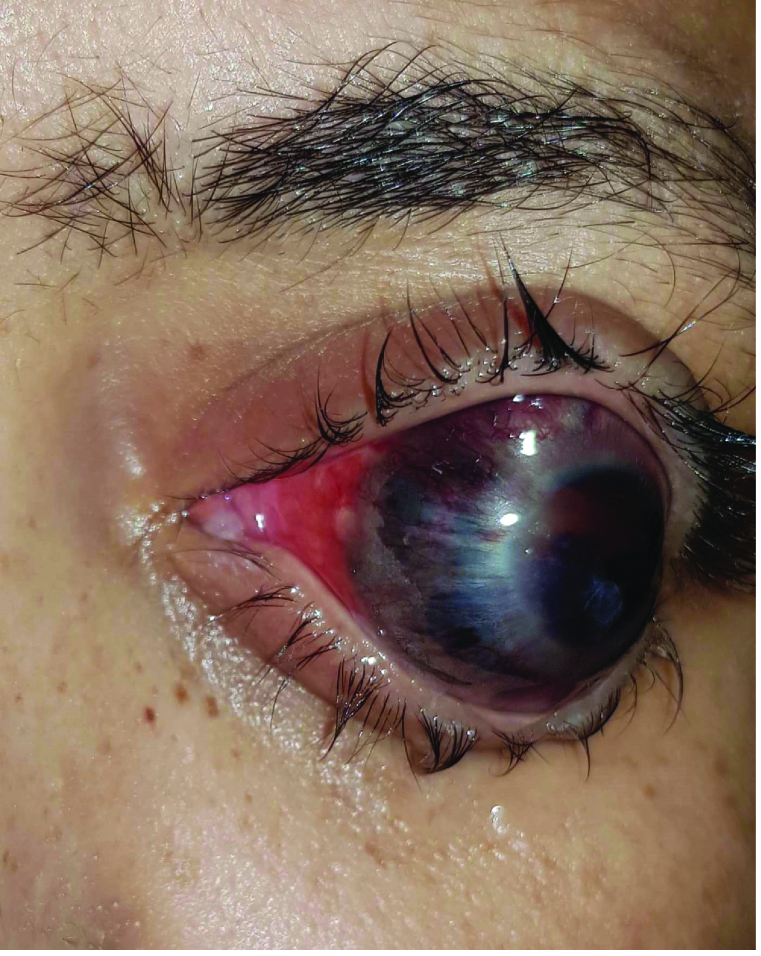
Protrusion and deviation of patient's eyeball.

At the time of her visit, the patient presented with an enlarged, buphthalmic eye [Figure 1]. There was scleral ectasia inferiorly. The cornea was cloudy with neovascularization of the iris. The anterior chamber was flat and the lens was pushed toward the cornea. The intraocular pressure was 28 mmHg.

**Figure 2:**
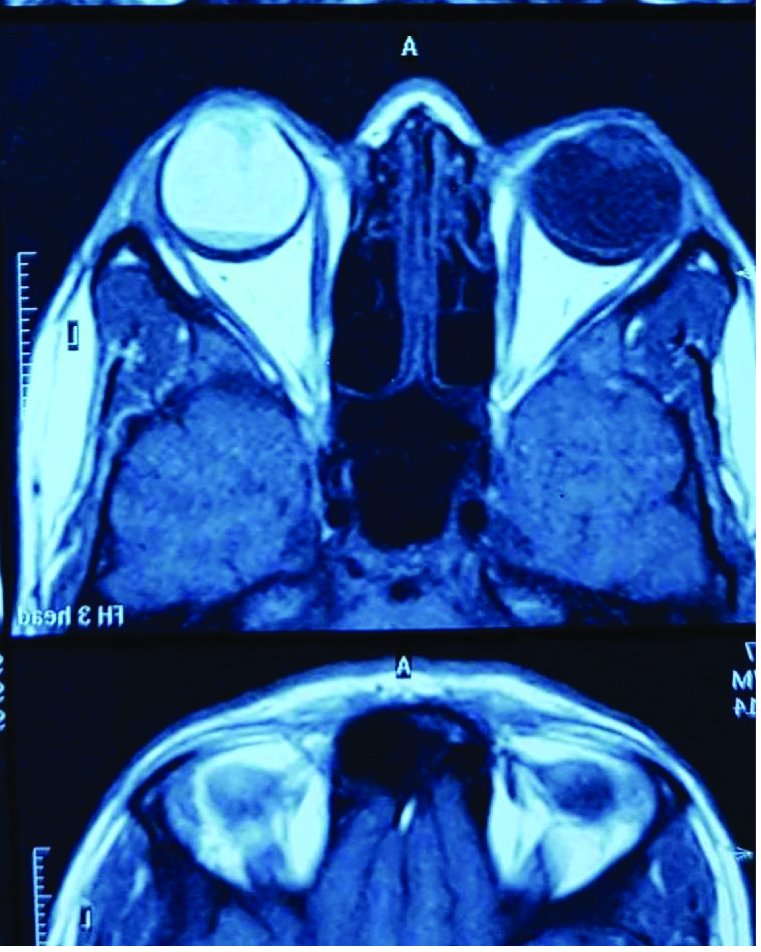
Magnetic Resonance Imaging: A mass occupying the lower part of the globe with a level of fluid in the back of the vitreous cavity without invasion of the optic nerve or the sclera.

A magnetic resonance imaging exam was performed. It revealed a mass occupying the lower part of the globe at the back of the vitreous cavity without invasion of the optic nerve or the sclera [Figure 2]. A decision was made to perform enucleation with histological examination of the mass.

**Figure 3:**
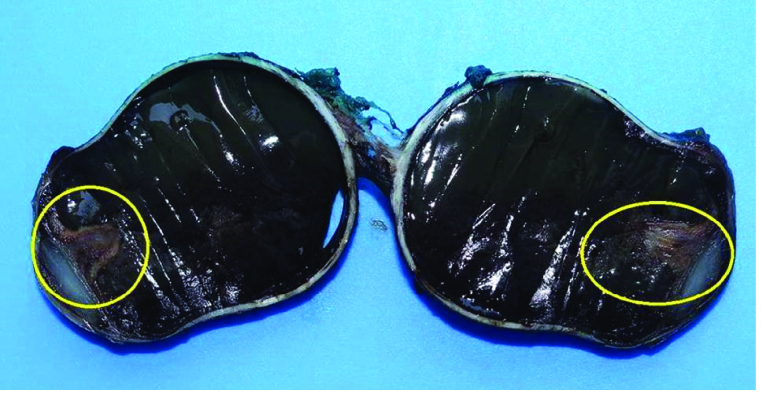
Gross specimen after fixation. Hemorrhagic solid mass.

At macroscopic examination, the eyeball weighed 12 gr and measured in diameter at 2.5 cm horizontally and 2 cm vertically. The optic nerve measured 0.5 cm in length and 0.4 cm in diameter. The anterior and posterior chambers were reduced in size. The lens measured 0.3 cm. There was a triangular-shaped tissue lesion that reached the posterior portion of the lens and appeared to originate from the ciliary body [Figure 3].

**Figure 4:**
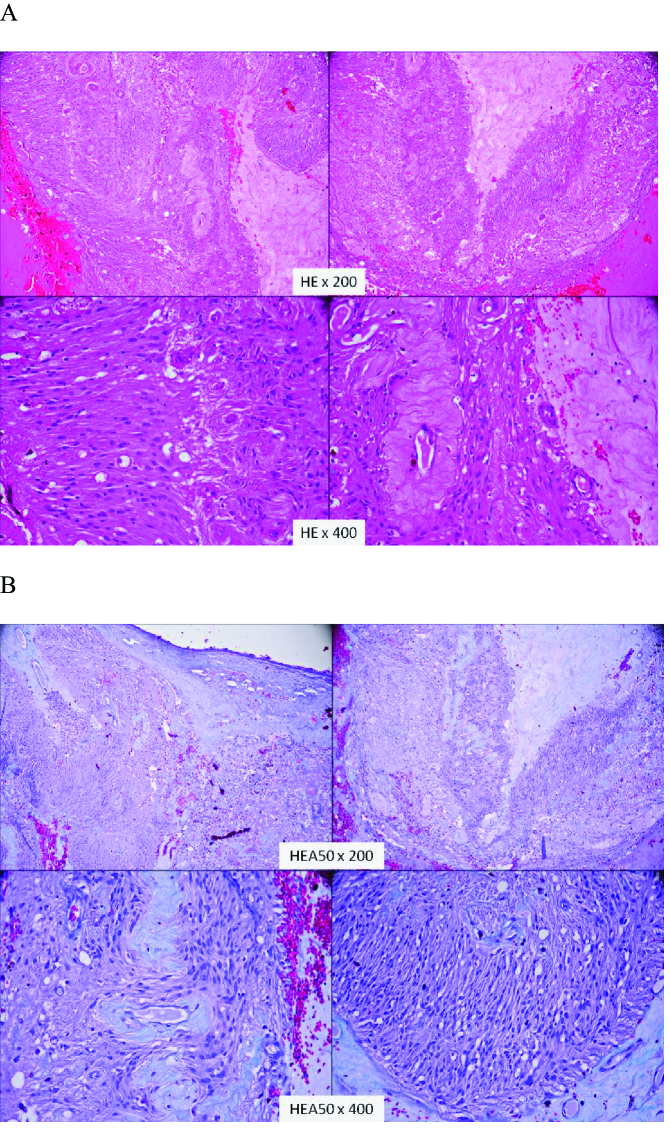
(a) Proliferation of mesenchymal monomorphic fusiform cells with Verocay body and Antoni type A/B patterns characteristic of schwannoma (HE × 200 + HE × 400). (b) Proliferation of mesenchymal monomorphic fusiform cells with Verocay body and Antoni type A/B patterns characteristic of schwannoma(HEA50 × 200 + HEA50 × 400).

The histological examination after HE and HEA50 staining showed proliferation of mesenchymal monomorphic fusiform cells with eosinophilic cytoplasm and small oval nuclei with a tendency toward palisading. Some parts of the tumor were hypercellular with a fascicular arrangement (Antoni A pattern); other parts were weakly cellular with a myxoid arrangement (Antoni B pattern). Several Verocay bodies and multiple hemorrhagic suffusions were present. Mitotic ﬁgures were very rare [Figures 4a and 4b].

**Table 1:** Ciliary body schwannoma: Literature review.


**Author**	**Year**	**Age**	**Sex**	**Histopathology**
Francois J^[10]^	1947	44	F	Antoni A + B
Donovan BF^[10]^	1956	68	F	Antoni A + B
Hogan MJ et al^[10]^	1962		Antoni A + B
Ferry AP^[10]^	1964		Antoni A + B
Racz M et al^[10]^	1973		Antoni A + B
Rosso R et al^[10]^	1983	40	F	Antoni A + B
Smith PA et al^[10]^	1987	30	F	Antoni A + B, Verocay bodies
Hufnagel TJ et al^[10]^	1988	57	F	Antoni A + B, Verocay bodies
Kuchle M et al^[10]^	1994	26	M	Antoni A + B, Verocay, S-100(+),Vimentin(+)
Pineda R et al^[10]^	1995	46	M	Antoni A + B: Verocay bodies, S-100(+),
Shields JA et al^[10]^	1997	70	F	Antoni A and S-100(+),
Thaller VT et al^[8]^	1998	28	F	Antoni A + B
Kim IT et al^[10]^	1999	39	F	Antoni A + B, Luse body, S-100(+), Vimentin (+)
Goto H et al^[3]^	2006	19	F	Antoni A + B, Luse body, S-100(+),
Kiratli H et al^[11]^	2010	4	M	Antoni A + B
	
	

**Figure 5:**
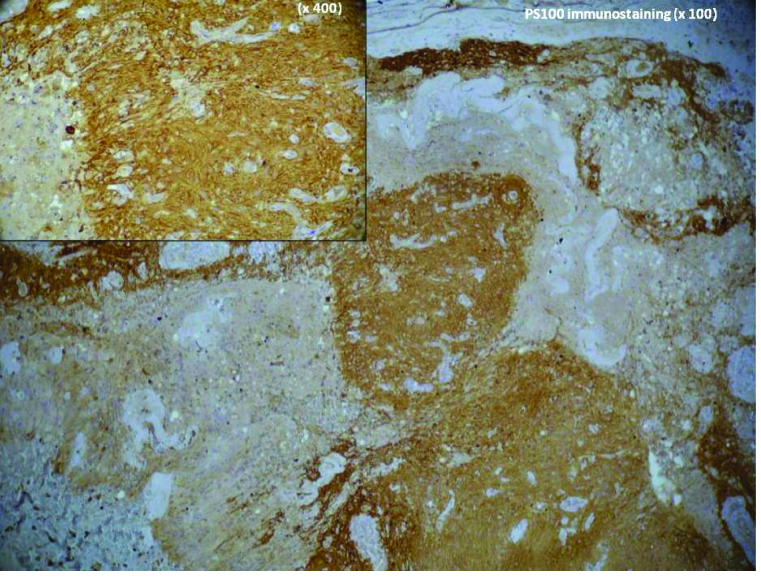
Immunohistochemistry staining showed that tumor cells were positive for S100 Protein (IHC × 200 + IHC × 400).

Immunohistochemistry staining showed that tumor cells were positive for PS100 and vimentin [Figure 5].

##  DISCUSSION

Schwannoma is a benign nerve sheath neoplasm of Schwann cells that occurs sporadically in any location of the body.^[1]^ Intraocular schwannomas are rare and most of them are not associated with neuroﬁbromatosis, malignant transformation of schawannoma may occur more frequently in the setting of type 1 neuroﬁbromatosis.^[2]^ It is hard to make the diagnosis of ciliary body neoplasm based only on clinical findings, even if a magnetic resonance imaging exam is applied.^[3]^

The MR imaging appearance of uveal schwannoma has been mentioned in just a few reports in ophthalmic literature, without accurate detailed descriptions. The few published reports describe a well-defined lesion getting enhanced after contrast administration.^[4,5,6]^

In most cases, enucleation was performed due to suspicion of malignant melanoma, but microscopic examination of the specimen revealed that the tumor was benign.^[7,8]^

Through clinical findings, there are several differential diagnoses of ciliary body schwannoma such as leiomyoma ciliary body melanoma, adenoma of the non-pigmented ciliary epithelium, and adult medulloepithelioma, but the hardest differential diagnosis is an unpigmented malignant melanoma which may be impossible to exclude with just clinical findings.^[9]^ Magnetic resonance imaging and noninvasive quantification of aqueous flare with the laser flare cell meter may also be helpful in the differential diagnosis of malignant and benign lesions. Fine needle biopsy and cytological examination is another alternative of differentiating ciliary body tumors. Rapid tumor growth, invasion of the anterior chamber and shifting of subretinal fluid represent some clinical signs which can suggest malignancy.^[9,10]^

Schwannomas have histological characteristic features: Antoni A/B area and Verocay bodies. Immunohistochemistry staining in our study showed that the tumor cells were positive for PS100 and vimentin.

In 1999, Kim and Chang reviewed the literature and identified 12 cases of ciliary body schwannomas.^[10]^ To our knowledge, just four more cases have been reported after that review, which makes a total of 16 reported cases.^[3,8,11]^ Enucleation was performed in most of these cases as malignant melanoma was suspected [Table 1].

In conclusion, the diagnosis of a benign schwannoma arising from the ciliary body was made based on the histopathology exam. This tumor is very rare. Even if the clinical signs are suspecting malignancy, complementary investigations including magnetic resonance imaging, noninvasive quantification of aqueous flare, and fine needle biopsy or local excision should be performed to first determine whether the tumor is benign or malignant to avoid overtreatment by enucleation.

##  Declaration of patient consent

The authors certify that they have obtained all appropriate patient consent forms. In the form the patient has given her consent for his images and other clinical information to be reported in the journal. The patient understand that her name and initial will not be published and due efforts will be made to conceal her identity, but anonymity cannot be guaranteed.

##  Financial Support and Sponsorship

None.

##  Conflicts of Interest

There are no conflicts of interest.
